# Associations between neighborhood stress and maternal sex steroid hormones in pregnancy

**DOI:** 10.1186/s12884-023-06043-0

**Published:** 2023-10-16

**Authors:** Megan C. Hansel, Hannah R. Murphy, Jessica Brunner, Christina Wang, Richard K. Miller, Thomas G. O’Connor, Emily S. Barrett, Zorimar Rivera-Núñez

**Affiliations:** 1 Department of Biostatistics and Epidemiology, Rutgers School of Public Health, Piscataway, NJ USA; 2 Department of Obstetrics and Gynecology, University of Rochester, Rochester, NY USA; 3 Clinical and Translational Science Institute, The Lundquist Institute at Harbor –UCLA Medical Center, Torrance, CA USA; 4 Departments of Psychiatry, Psychology, Neuroscience, University of Rochester, Rochester, NY USA

**Keywords:** Neighborhood stress, Pregnancy, Hormones, Testosterone, Estrogen

## Abstract

**Background:**

Neighborhood stressors (e.g., crime and deprivation) have been associated with adverse pregnancy outcomes including preterm birth and low birth weight. A potential mechanism is disruption of maternal endocrine pathways. While stress hormones (e.g., cortisol) have received much attention, other relevant hormones, including sex steroids, have been overlooked.

**Methods:**

Pregnant women in the Understanding Pregnancy Signals and Infant Development (UPSIDE) study contributed biospecimens, questionnaires, and medical record data (n = 262). In each trimester, maternal serum total testosterone [TT], estrone, estradiol, and estriol were measured using LC/MS-MS and serum free testosterone was measured by equilibrium dialysis. In the third trimester, participants reported on neighborhood stress over the last year through the validated City Stress Inventory. We examined two subscales: 11-item neighborhood disorder (e.g., vacant buildings, crime) and 7-item exposure to violence (personal experiences of violence). Composite scores were calculated and examined categorically (quartile (Q) for neighborhood disorder and any/none for exposure to violence). We fitted linear mixed models examining associations between neighborhood stressors and sex steroid hormones across pregnancy as well as trimester-specific linear regression models, all adjusting for confounders. Secondarily, we stratified by fetal sex. Results are presented as percentage change (∆%) and 95% confidence interval (CI) in hormones.

**Results:**

Most participants (73%) reported one or more exposures to neighborhood disorder; 22% reported any exposure to violence. In adjusted models, neighborhood disorder was associated with higher TT across pregnancy (Q2: %∆= 37.3, 95%CI: 13.2, 66.5; Q3: %∆= 22.2, 95%CI: 1.2, 47.5; and Q4: %∆= 25.7, 95%CI: 1.6, 55.3), with the strongest associations observed in the third trimester (Q2: %∆= 38.0, 95%CI: 10.6, 72.1; Q3: %∆= 29.2, 95%CI: 4.4, 59.9; and Q4: %∆=33.4, 95%CI: 4.9, 69.6). In stratified models, neighborhood disorder was associated with higher TT among women carrying male fetuses (%∆ range: 48.2–84.8). Exposure to violence was not associated with any hormones.

**Conclusion:**

Neighborhood disorder is associated with higher maternal testosterone levels, which may have implications for maternal and child health. Additional research is needed to understand the mechanisms by which neighborhood stress impacts endocrine physiology.

**Supplementary Information:**

The online version contains supplementary material available at 10.1186/s12884-023-06043-0.

## Introduction

Extensive evidence demonstrates that the characteristics of the neighborhoods in which people reside can influence their health, including risks of cardiovascular disease, obesity, sleep disorders, anxiety and depression [1–4]. Studies of neighborhoods and health have additionally informed our understanding of spatial distributions of disease and health disparities. One area of increasing interest is how neighborhood social and physical characteristics may contribute to perinatal health including pregnancy outcomes [5–8]. For example, in a study of women in Chicago, participants living in neighborhoods with high levels of physical disorder (i.e., abandoned buildings, defaced property, building conditions), had a higher prevalence of hypertensive disorders of pregnancy compared to participants living in neighborhoods with low levels of physical disorder [9]. In a Swedish study, pregnant people living in a severely socially deprived area (defined as having low socioeconomic resources and high rates of unemployment, diverse ethnicities, overcrowding, segregation, criminality, and norm-breaking behaviors) had 90% higher odds of having an extremely preterm birth compared to pregnant people living in an area without deprivation [10]. These studies show similar results across different geographical areas and populations (Chicago vs. Sweden). To date, little research has examined the mechanisms by which neighborhood stressors may disrupt perinatal physiology, leading to adverse birth outcomes [11].

One possibility is that neighborhood features such as crime and disorder are sources of individual-level psychosocial stress, which has been associated with adverse pregnancy outcomes [12, 13]. For example, in pregnant women residing in Philadelphia, positive associations were observed between neighborhood level of crime and perceived stress [14]. Individual-level perceived stress can influence pregnancy physiology, leading to hormonal and neuroendocrine changes that may adversely impact maternal and/or fetal health [15, 16]. While the vast majority of research focuses on the hypothalamic-pituitary-adrenal (HPA) axis as the primary pathway underlying response to stressors [17–19], there are fewer studies examining other potentially relevant hormonal pathways, such as the hypothalamic-pituitary-gonadal (HPG) axis, which may also be sensitive to stressors and is known to engage in cross-talk with the HPA axis [20]. For instance, in animal models, prenatal stress disrupts the normal surge of testosterone in the developing male [21–23]. While human studies examining the relationship between prenatal stress and hormones produced by the HPG axis are limited, there is evidence linking prenatal stress to anogenital distance (AGD), a biomarker of the prenatal hormonal milieu that is thought to reflect prenatal androgen activity [24]. For example, one study found that couples who reported experiencing more stressful life events in pregnancy had female offspring with a longer, more androgenized AGD compared to couples who reported less stressful life events [25]. Similarly, in a study of Finnish adolescents who gestated during and after the Chernobyl nuclear disaster (considered a stressful life event), exposure to the stressor was associated with increased testosterone levels in female offspring [26]. The current study adds to prior research on environmental sources of stress and sex steroids of the HPG axis.

Given their critical role in pregnancy, including support of fetal growth and maturation, disruptions of maternal sex steroid hormones, such as those due to stressors, may be detrimental to the course of pregnancy. For example, many studies have linked maternal estrogen and testosterone in pregnancy to birth outcomes including birth weight and length [27–31]. Further, reproductive conditions characterized by altered hormone levels such as polycystic ovary syndrome have been associated with birth outcomes including preterm birth and small for gestational age [32–34]. Maternal sex steroid hormone levels may also impact a child’s long-term health and behavioral outcomes, as the prenatal hormonal milieu may “program” child development [35–37].

Although several prior studies have examined individual level factors impacting maternal sex steroid levels [38, 39], to our knowledge, no prior studies have assessed how neighborhood stressors may impact these important hormones during pregnancy. To that end, the aim of this study is to examine associations between neighborhood stress and maternal sex steroid hormone concentrations (testosterone and estrogens) across pregnancy using data from a longitudinal pregnancy cohort.

## Methods

### Study overview and population

From December 2015 to April 2019, n = 326 participants were enrolled in the Understanding Pregnancy Signals and Infant Development (UPSIDE) cohort study during their first trimester of pregnancy from outpatient obstetric clinics affiliated with Rochester University Medical Center [40]. Inclusion criteria included: <14 weeks gestation, age 18 or older, singleton pregnancy, no known substance abuse problems or a history of psychotic illness, and the ability to communicate in English. Women with major endocrine disorders (e.g., polycystic ovary syndrome), high-risk pregnancies, or significant obstetric problems at the time of enrollment were excluded. The current analysis was restricted to women with hormone data, at least one complete neighborhood stress subscale (disorder or violence), and complete covariate data. Participants engaged in face-to-face visits during each trimester of their pregnancy, during which biospecimens were collected and questionnaires were administered. The UPSIDE study was approved by the University of Rochester School of Medicine and Dentistry Institutional Review Board and the Rutgers University Institutional Review Board and all participants provided informed consent prior to engaging in study activities.

### Measures

#### Neighborhood stress

Neighborhood stress was measured through a validated City Stress Inventory (CSI) questionnaire that was administered to participants during their third trimester visit [41]. The CSI contains 18 total questions and is divided into 2 subscales- neighborhood disorder (e.g., vacant buildings, crime) which consists of 11 questions and exposure to violence (personal experiences of violence) which contains 7 questions. Sixteen items queried how often different neighborhood-related stressors occurred in the last year, including time while pregnant, with response choices including never, once, a few times, or often. The remaining two items quantified neighborhood disorder/poverty and included answer choices for none, some, about half, or most. Responses were scored on a scale from 1 to 4, with 1 corresponding to never/none and 4 corresponding to often/most. The response values from each subscale were totaled to calculate composite scores for neighborhood disorder (range 11–44) and violence (range 7–28), with lower scores indicating less neighborhood disorder or violence. Given the skewed distribution of responses, we categorized neighborhood disorder scores in quartiles and neighborhood violence as any reported exposure versus none.

#### Maternal sex steroid hormones

Blood samples were collected in each trimester, then processed and frozen at -80°C. Serum aliquots from each trimester were shipped on dry ice to the Endocrine and Metabolic Research Laboratory at the Lundquist Institute at Harbor-UCLA Medical Center. We examined five hormones- estrone (E1), estradiol (E2), estriol (E3), total testosterone (TT), and free testosterone (FT). E1, E2, E3, and TT were assayed using gold standard liquid chromatography with tandem mass spectrometry (LC-MS/MS) protocol with slight modifications to the runtime and system parameters [36, 42–44]. A Shimadzu HPLC system (Columbia, MD) and an Applied Biosystems API5500 LC-MS/MS (Foster City, CA) with a Turbo-Ion-Spray source using positive mode was used for TT assessment. The Shimadzu HPLC system (Columbia, MD) was also used for estrogen assessment, with a triple quadrupole mass spectrometer (API5000 LC-MS/MS, Foster City, CA). FT, the unbound and biologically active fraction of testosterone, was measured through equilibrium dialysis using labeled testosterone [45]. In the first trimester, forty-nine participants had E3 values below the limit of detection (LOD). Observations below the LOD were imputed by assigning the value of LOD/(square root of 2) [46].

#### Demographic covariates

Demographic information was generally collected at baseline using questionnaires. Continuous variables included gestational age at hormone measurement, maternal age, and early pregnancy body mass index (body mass index [BMI] in kg/m^2^). BMI was calculated using height and weight from the earliest first trimester visit, because capturing participant’s actual pre-pregnancy BMI is challenging in a pregnancy cohort. However, research shows that this is a suitable substitute, as maternal weight in the first trimester is typically within 1-2 kg of pre-pregnancy weight [47]. Categorical variables included highest maternal education level (less than high school, high school; some college, bachelors; and post graduate degree), insurance status (Medicaid: yes or no), sex of fetus (male or female), and parity (0 or ≥ 1). We additionally included maternal race (Non-Hispanic White, Non-Hispanic Black, other) and maternal ethnicity (Non-Hispanic/Non-Latina or Hispanic/Latina) as proxies for systemic racism which may influence neighborhood of residence and contribute to physiologic dysregulation, including alterations in pregnancy physiology [48–50].

## Statistical analysis

Summary statistics (mean, standard deviation, median, quartiles, range) were assessed for continuous variables; frequencies and percentages were calculated for categorical variables. Preliminary analysis showed the sex steroid hormones were not normally distributed, so they were natural log transformed for all subsequent analyses. The correlation among hormones was assessed by Pearson correlation analysis. We fitted two sets of models: one with neighborhood disorder as the main exposure and the other with exposure to violence as the main exposure. We first conducted unadjusted linear regression analyses examining associations between the two neighborhood stress exposure measures and the five individual hormone measures in each trimester. Potential confounders were selected *a priori* from existing literature and directed acyclic graphs (Supplemental Fig. 1). From the set of potential covariates described above, those that appreciably changed the beta estimates by 10% or more from the crude associations were included in final adjusted models. Final multivariable linear regression models were adjusted for maternal age, early pregnancy BMI, gestational age at visit, maternal race, maternal ethnicity, highest maternal education, Medicaid in pregnancy, parity, and infant sex. Our primary models capitalized on the repeat hormone measurements using linear mixed models (LMMs), with random intercepts. To adjust for variation in gestational age at sample collection, models included a fixed effect for neighborhood stressors, a smooth function for gestational age (using gestational age deviation), and a random effect for individual participants within an unstructured correlation matrix since we did not have *a priori* assumptions for correlation patterns. Secondarily, we fit models examining associations with hormone concentrations in each individual trimester, prioritizing third trimester hormone concentrations which were measured contemporaneously with the neighborhood stress data. Because stress may impact male and female fetuses differently [51], we repeated all analyses above stratifying by fetal sex. Lastly, we conducted sensitivity analyses excluding participants who moved during pregnancy. Results were back-transformed to obtain estimates representing percentage change (∆%) and 95% confidence interval (CI) in hormones. P-values < 0.05 were considered significant. Analyses were performed in SAS 9.4.

## Results

Eight participants did not have hormone data, 42 did not have any neighborhood stress data (both the disorder and violence subscales were missing), and 14 did not have complete covariate data bringing the final sample to 262 participants. Participants were on average 29.2 ± 4.6 years old with an early pregnancy BMI of 28.0 ± 7.2 kg/m² (Table 1). The majority were Non-Hispanic White (63.7%), had a college/bachelor’s degree (38.9%), did not use Medicaid during pregnancy (55%), did not relocate during pregnancy (90.8%) and had a prior live birth (67.9%), with 9.9% reporting Hispanic ethnicity. Overall, 73.3% of participants reported any neighborhood disorder and 22.3% reported neighborhood violence.

**Table 1:** Characteristics of UPSIDE cohort participants (n = 262)

Characteristics	Mean ± SD	Min, Max
Maternal Age	29.19 ± 4.55	18, 41
Early pregnancy BMI (kg/m²)	27.99 ± 7.22	15.31, 49.09
GA at Trimester 1 Visit (weeks)	12.24 ± 1.28	6.14, 14.43
GA at Trimester 2 Visit (weeks)	21.17 ± 1.76	18.14, 29.57
GA at Trimester 3 Visit (weeks)	31.34 ± 1.91	28.14, 39.00
	**Frequency**	**Percentage**
Maternal Race		
Non-Hispanic White	167	63.74%
Non-Hispanic Black/African American	62	23.66%
Other^1^	33	12.60%
Maternal Ethnicity		
Hispanic or Latino	26	9.92%
Non-Hispanic or Non-Latino	236	90.08%
Highest Maternal Education		
High School or Less	96	36.64%
Some College, Bachelors	102	38.93%
Postgraduate	64	24.43%
Medicaid in Pregnancy		
Yes	118	45.04%
No	144	54.96%
Relocated in Pregnancy		
Yes	24	9.16%
No	238	90.84%
Multiparous		
Yes (> 1)	178	67.94%
No (0)	84	32.06%
Fetal Sex		
Male	132	50.38%
Female	130	49.62%
**Neighborhood Stress**	**Frequency**	**Percentage**
Neighborhood Disorder^2^ (n = 258)		
Q1 (score = 11)	69	26.74%
Q2 (score = 12–13)	63	24.42%
Q3 (score = 14–17)	71	27.52%
Q4 (score = 18–42)^2^	55	21.32%
Exposure to Violence (n = 260)		
None (score = 7)	202	77.69%
Any (score > 7)	58	22.31%

Abbreviations: SD- Standard Deviation, BMI- Body Mass Index, GA- Gestational Age

^1^Other includes Asian, American Indian/Alaska Native, and more than one race

^2^Highest potential score was 44. Highest participant response was 42

Median concentrations of maternal serum estrogens increased steadily across pregnancy while TT and FT concentrations were similar across trimesters (Table 2). Among the estrogens, E1 and E2 were strongly correlated (r: 0.81, 0.78, 0.77), E1 and E3 were weakly correlated (r: 0.19, 0.29, 0.18), and E2 and E3 were moderately correlated (r: 0.29, 0.45, 0.41) in trimesters one, two, and three, respectively. Among the androgens, TT and FT were weakly correlated in trimesters 1 (r: -0.13), 2 (r: -0.04), and 3 (r: 0.11). Androgens and estrogens were weakly correlated in all trimesters (Supplemental Fig. 2).

**Table 2:** Descriptive statistics of maternal sex steroid hormone concentrations

	Min	Q1 (25%)	Median	Q3 (75%)	Max	Mean ± SD
**Trimester 1 (n = 260)**						
TT (ng/dl)	8.80	36.55	59.55	87.20	303.00	70.23 ± 45.76
FT (ng/dl)	0.23	0.48	0.56	0.66	1.17	0.58 ± 0.16
E1 (pg/ml)	124.0	562.0	937.0	1425.0	5640.0	1137.12 ± 873.36
E2 (pg/ml)	356	1160	1605	2325	5940	1831.65 ± 951.67
E3 (pg/ml)	35.36	87	228.5	472.5	1470	303.68 ± 265.52
**Trimester 2 (n = 262)**						
TT (ng/dl)	6.9	42.6	63.1	97.8	413.5	78.34 ± 54.42
FT (ng/dl)	0.19	0.43	0.49	0.56	0.93	0.50 ± 0.13
E1 (pg/ml)	423	2210	3480	5180	20,500	4146.47 ± 2904.17
E2 (pg/ml)	1750	4650	5895	7880	18,500	6407.02 ± 2708.38
E3 (pg/ml)	799	2330	3090	3880	7700	3182.35 ± 1204.46
**Trimester 3 (n = 260)**						
TT (ng/dl)	9.60	36.75	62.95	101.00	349.50	77.35 ± 57.12
FT (ng/dl)	0.20	0.43	0.50	0.58	1.04	0.51 ± 0.14
E1 (pg/ml)	751	3625	5855	8135	37,700	6748.17 ± 5116.65
E2 (pg/ml)	2380	8960	11,050	14,300	31,900	11943.73 ± 4786.63
E3 (pg/ml)	1030	5140	6605	8370	26,300	7183.04 ± 3385.91

Abbreviations: Q1- 25% Quantile, Q3- 75% Quantile, SD- Standard Deviation, TT- Total Testosterone, FT- Free Testosterone, E1- Estrone, E2- Estradiol, E3- Estriol

**Fig. 1:**
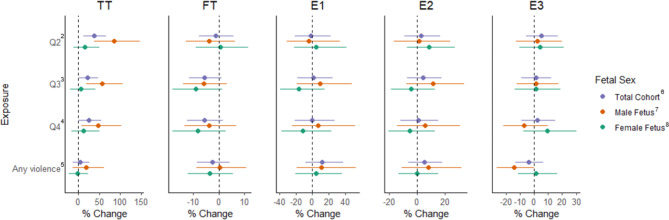
Adjusted^1^ LMMs examining neighborhood stress and hormone concentrations in full cohort and by fetal sex. ^1^Adjusted for trimester, maternal age, early pregnancy BMI, gestational age deviation, fetal sex, parity, maternal race, maternal ethnicity, highest maternal education level, and Medicaid in pregnancy. *Stratified models not adjusted for fetal sex. ^2^Neighborhood disorder score = 12–13. ^3^Neighborhood disorder score = 14–17. ^4^Neighborhood disorder score = 18–42. Neighborhood disorder reference category is Q1 where score = 11. ^5^Any violence score > 7. Reference is no violence where score = 7. ^6^Neighborhood disorder (n = 258) and exposure to violence (n = 260). ^7^Neighborhood disorder (n = 130) and exposure to violence (n = 131). ^8^Neighborhood disorder (n = 128) and exposure to violence (n = 129)

In our primary adjusted LMMs, compared to participants in the lowest quartile of neighborhood disorder, those who experienced greater neighborhood disorder had higher concentrations of TT across pregnancy (quartile 2: %∆= 37.3, 95%CI: 13.2, 66.5; quartile 3: %∆= 22.2, 95%CI: 1.2, 47.5; and quartile 4: %∆= 25.7, 95%CI: 1.6, 55.3) (Fig. 1, Supplemental Table 1). No statistically significant associations were found between neighborhood disorder and the 4 remaining hormones. In LMMs examining exposure to violence, we observed crude associations between exposure to violence and higher TT as well as E2, however associations were attenuated and non-significant after adjustment for covariates; no other hormones were associated with exposure to violence (Fig. 1, Supplemental Table 1).

In LMMs stratified by fetal sex, associations between neighborhood disorder and TT were positive in both groups, but were stronger among women carrying male fetuses (Fig. 1, Supplemental Table 2). For example, compared to participants in the lowest quartile of neighborhood disorder, women carrying male fetuses in the second quartile of neighborhood disorder had 84.8% higher TT (95%CI: 37.8, 148.0), while women carrying female fetuses in the second quartile of disorder had 15.2% higher TT across pregnancy (95%CI: -11.5, 49.9).

As a secondary approach to evaluate differences in associations across pregnancy, we fit linear regression models examining neighborhood stress and maternal sex steroid hormones in each individual trimester. Overall, the strongest associations were detected for neighborhood disorder and third trimester TT concentrations (Table 3**)**. In adjusted models, compared to participants experiencing the lowest levels of neighborhood disorder, women reporting higher levels of neighborhood disorder had higher concentrations of third trimester TT (quartile 2: %∆= 38.0, 95%CI: 10.6, 72.1; quartile 3: %∆= 29.2, 95%CI: 4.4, 59.9; and quartile 4: %∆= 33.4, 95%CI: 4.9, 69.6).

**Table 3:** Adjusted^1^ linear regression models examining neighborhood stress and maternal hormone concentrations in the third trimester

	Total Testosterone (TT, ng/dl)	Free Testosterone (FT, ng/dl)	Estrone (E1, pg/mL)	Estradiol (E2, pg/mL)	Estriol (E3, pg/mL)
	%Δ (95% CI)	%Δ (95% CI)	%Δ (95% CI)	%Δ (95% CI)	%Δ (95% CI)
**Neighborhood Disorder** ^**2**^ **(n = 256); REF = Q1**					
Q2	**37.99** **(10.64, 72.10)**	0.30 (-9.33, 10.95)	-5.86 (-25.43, 18.84)	2.52 (-9.63, 16.30)	3.43 (-9.63, 18.39)
Q3	**29.18** **(4.39, 59.87)**	-8.12 (-16.64, 1.27)	7.81 (-13.90, 34.98)	8.54 (-3.90, 22.59)	1.91 (-10.53, 16.09)
Q4	**33.39** **(4.92, 69.59)**	-4.16 (-14.11, 6.95)	12.96 (-12.31, 45.51)	6.24 (-7.38, 21.85)	-0.35 (-13.95, 15.40)
**Exposure to Violence** ^**3**^ **(n = 258); REF = none**					
Any	11.20 (-9.90, 37.25)	-2.83 (-11.66, 6.88)	16.36 (-6.49, 44.78)	6.80 (-5.35, 20.51)	-3.39 (-14.99, 9.81)

Abbreviations: %Δ-Percent Change, CI- Confidence Interval. ^1^Models adjusted for maternal age, early pregnancy BMI, gestational age at blood sample, fetal sex, parity, maternal race, maternal ethnicity, highest maternal educational level, and Medicaid in pregnancy. ^2^Q1 score = 11, Q2 score = 12–13, Q3 score = 14–17, Q4 score = 18–42. ^3^None score = 7, any score > 7. Bolded entries indicate p < 0.05

In trimesters one and two, women in the second quartile of neighborhood disorder (score = 12–13) had higher concentrations of TT (trimester 1: %∆= 35.1, 95%CI: 9.6, 66.6; trimester 2: %∆= 40.4, 95%CI: 13.4, 73.9) compared to women with the lowest levels of neighborhood disorder (Supplemental Table 3). No trimester-specific associations between neighborhood disorder and estrogens were observed (Table 3, Supplemental Table 3). In unadjusted models, mothers reporting exposure to violence had significantly higher E2 concentrations in the first and third trimesters **(not shown)**, however associations were attenuated after adjustment for covariates and no additional trimester-specific associations between neighborhood violence and sex steroid concentrations were observed (Table 3, Supplemental Table 3). Results of trimester-specific models stratified by fetal sex were similar to those of the LMMs, with stronger, significant associations between neighborhood disorder and TT observed in all trimesters in participants carrying males compared to females (**not shown**). For both the adjusted LMMs and trimester-specific linear regression models, associations were robust to the exclusion of the 24 participants (9.2%) who relocated during pregnancy, with the direction and magnitude of the estimates remaining similar (Supplemental Table 4, Supplemental Table 5).

## Discussion

In this analysis of neighborhood stressors and sex steroid hormones measured across pregnancy, higher neighborhood disorder was associated with higher maternal testosterone concentrations across pregnancy, these associations were stronger among women carrying male fetuses and grew stronger in late pregnancy. No associations were observed in relation to estrogen concentrations, nor was neighborhood violence associated with any sex steroid hormone concentrations. To our knowledge, this is the first epidemiological study to report that neighborhood-level stressors may affect HPG axis activity during pregnancy.

The association between prenatal stress and sex steroid hormone activity has been demonstrated in numerous animal studies. Consistent with our results, these studies provide evidence that stress is associated with disrupted androgen levels, with notable differences by offspring sex [23, 52]. However these studies generally report that prenatal stress disrupts the male fetus’ testosterone surge [21–23], results that are further supported by evidence that compared to controls, the offspring of prenatally stressed dams have a reduced anogenital distance, indicative of less prenatal androgen exposure [53]. In contrast to those results, our study found maternal neighborhood stress was associated with higher testosterone levels, however a key distinction is that animal studies have generally examined testosterone levels in the offspring, while our study captures circulating maternal hormone levels, which reflect production by mother, placenta, and fetus. While a larger body of literature focuses on testosterone, associations between prenatal stress and estrogens have also been noted. Specifically, one study found that rats who were exposed to environmental prenatal stress had lower levels of estradiol compared to control rats [54]. To date, few human studies have examined maternal stress in pregnancy in relation to estrogens. Additional studies are required to provide greater insight into individual-level psychosocial measures and affective symptoms in relation to HPG axis activity during human pregnancy.

Neighborhood-level stressors may contribute to endocrine dysregulation through their impacts on individual-level experiences of stress and affective symptoms. The connection between neighborhood and individual stress has been well-studied both in pregnant and non-pregnant individuals. For example, in a study of 1,309 pregnant women in Philadelphia, those living in a neighborhood with high violent crime, assessed via public violent crime data, had 1.38 times higher odds of having high perceived stress compared to those living in a neighborhood with low violent crime [14]. Additional studies have identified maternal stress as a partial mediator in associations between neighborhood factors and outcomes such as maternal depression and birth weight, demonstrating how neighborhood characteristics may affect maternal and child outcomes through stress [55, 56]. Impacts of neighborhood characteristics on perceived stress and affective symptoms have also been shown in non-pregnant people. For example, in one study adolescents who perceived their neighborhood to be unsafe were 2.4 times more likely to report psychological distress compared to those who perceived their neighborhood to be safe [57]. Similar results have been demonstrated in older adults. In a population of mostly low-income, older African Americans, those who reported their neighborhoods to be safer and more socially cohesive had lower levels of perceived stress [58]. Thus, there is strong evidence to suggest that neighborhoods are important predictors of individual-level measures of mental health.

In pregnant people, androgens are produced by the mother (ovary and adrenal cortex), placenta, and the fetus (gonads and adrenal cortex) [59, 60]. One or more of these physiological sources of androgens may be responsive to stressors, but maternal circulating hormones alone are insufficient to determine the source. One biologically plausible explanation for our results suggesting higher testosterone associated with greater neighborhood disorder is upregulation of adrenal androgen activity. If neighborhood stress results in greater activation of the HPA axis, as suggested by some studies [61, 62], higher adrenocorticotropic hormone (ACTH) production may stimulate synthesis of adrenal androgens, including testosterone and dehydroepiandrosterone [63, 64]. In our study, associations between neighborhood disorder and testosterone strengthened across pregnancy, which may be the result of the changing nature of the HPA axis across gestation resulting from the exponential increase in placental corticotropin releasing hormone (CRH) production as pregnancy progresses [65, 66]. As placental CRH gets released into maternal circulation, it can stimulate ACTH, which in turn may lead to greater testosterone being released in later trimesters [66]. While one might expect higher stress to alter hormone activity in a dose-response manner, we observed that the lower quartiles of neighborhood disorder had stronger associations with testosterone than the higher quartiles. While we are unsure of what may be driving this interesting pattern, one potential explanation is that chronic stress may lead to habituation and blunting of the stress response, as is seen in the case of flattened diurnal cortisol levels following chronic stress or trauma [67]. Given known interactions between the HPA and HPG axes and that in women, 50% of androgens are of adrenal origin [68, 69], it is possible that high chronic stress may also blunt testosterone production in women, while lower levels of stress may result in greater activation.

We also noted that the relationship between neighborhood disorder and testosterone levels were stronger in mothers carrying male fetuses. During gestation, the fetal testes are a major source of testosterone production [70] and the fetal testosterone surge is largely responsible for sexual differentiation [71]. The extent to which fetal testicular testosterone production is reflected in maternal circulating concentrations is unclear; some studies have shown differences in maternal testosterone by fetal sex, while others have not [72, 73]. Some evidence from animal models suggests that maternal stress may dampen testosterone production in the fetal testes [21], while our results may be reflective of heightened testicular testosterone production given the observed differences by fetal sex in stratified models. Additionally, other human studies have shown evidence that male fetuses may be more sensitive to fetal insults. For example, a study in the Philippines found that maternal evening cortisol levels was a significant predictor of birth weight for male, but not female, offspring [74]. However, a study in Israel showed that female fetuses were more sensitive to prenatal stress in relation to birth outcomes [75]. Our results support previous research demonstrating increased sensitivity among male fetuses, as we show that women carrying male fetuses have stronger associations between neighborhood disorder and maternal testosterone than women carrying female fetuses. Lacking technologies to assess human fetal hormone activity in utero for research purposes, future research on maternal stressors and androgen-sensitive endpoints, such as anogenital distance, may shed light on this issue.

Better understanding the potential mechanisms by which maternal stressors (on both the individual and neighborhood levels) impact pregnancy physiology is critical as previous literature suggests that maternal stress may lead to adverse birth outcomes such as low birth weight and preterm birth [56, 76]. For example, a study by Gilles et al., used a composite measure of 6 different psychological questionnaires to measure prenatal stress during the third trimester and found that maternal prenatal stress was associated with a significant reduction in birth weight (− 217 g), birth length (− 1.2 cm), and head circumference (− 0.8 cm) [77]. Another study observed that compared to women who gave birth at term, women who had a preterm birth were 2.15 times more likely to have experienced maternal stress during pregnancy [78]. Additionally, neighborhood characteristics are associated with adverse pregnancy outcomes. Masi et al., used census tract level data to measure neighborhood economic disadvantage and violent crime rates and found they were each associated with increased odds of low birth weight for White, Black, and Hispanic children. The impact of neighborhood characteristics also varied by the racial composition of the census tract, as economic disadvantage and violent crime rates both increased as the proportion of Black and Hispanic residents increased [79]. Neighborhood economic disadvantage has also been associated with preterm birth. For example, Black women living in a wealthier census tract had a reduced risk of preterm birth compared to Black women living in a poorer census tract [80]. Similarly, research shows that residing in a neighborhood with favorable conditions (determined by social, environmental, and educational factors) is positively associated with birth length and weight [81]. An additional mechanism to consider is epigenetic modifications. Research shows that prenatal neighborhood conditions including poverty, public assistance, and healthy food deficiency are associated with epigenetic changes such as gestational epigenetic age deceleration [82]. Stress has also been associated with epigenetic alteration, as a meta-analysis found that maternal stress was correlated with DNA methylation [83]. Overall, maternal stress and neighborhood characteristics have an impact in population health and this impact may have long-term consequences particularly for *in utero* exposures. Some evidence from non-pregnant women suggests that interventions to lower stress may also lower testosterone levels [84] and in non-pregnant women, neighborhood characteristics such as increased walkability have also been associated with reduced testosterone levels [85]. Implications of altered prenatal testosterone activity on perinatal outcomes remain unknown and need further study.

In this first study to examine the association between neighborhood stress and maternal sex steroid hormone levels, an important strength was the measurement of maternal hormone levels at each trimester, which enabled us to examine the associations across pregnancy as well as within each trimester. Additionally, hormone analysis was done using gold standard of LC/MS-MS and equilibrium dialysis for free testosterone. Most studies to date have used older, less sensitive immunoassay techniques, however LC/MS-MS is essential for measuring androgens in pregnancy given their low concentrations as well as the potential for cross-reactivity with other steroids in immunoassay platforms [86–88]. Another strength was our use of the validated CSI questionnaire to assess neighborhood stressors. Maternal perceptions of neighborhood stress (such as those measured by the CSI) may be informative when considering impacts on maternal physiology. Our study sample was relatively diverse in socioeconomic status (e.g., participant’s education ranged from high school to postgraduate) and reflected women living in a range of Upstate, NY neighborhoods including urban, suburban, and even rural areas. At the same time, we note some limitations. Neighborhood stress was measured at a single time point in the third trimester, while maternal hormones were measured in the first, second, and third trimester. However, questions on the City Stress Inventory referred to neighborhood environment in the prior year. Additionally, like other research indicating that most women do not move during pregnancy [89], only 24 (9.2%) of our sample reported relocating during pregnancy and sensitivity analyses excluding these participants showed similar results, demonstrating the robustness of our findings. Nevertheless, aside from the models examining neighborhood stress and third trimester hormones, we cannot establish causality due to the timing of the hormone measurements preceding the exposure measurement. Another limitation of our study is that a small number of participants reported exposure to violence, which may have affected our power to examine this association. It is possible that an association might have been detected if there was a larger sample with more exposure to violence. Similarly, due to smaller samples in some strata, we did not examine associations by race/ethnicity or socioeconomic strata and it is possible that the impact of neighborhoods on pregnancy physiology may differ by sociodemographic factors. Additional work in large, diverse samples is warranted. Finally, no objective measures of stress such as crime data were used, but would complement our self-reported neighborhood data.

## Conclusion

Our findings suggest that greater exposure to neighborhood disorder is associated with higher levels of testosterone across pregnancy, particularly among mothers carrying male fetuses. Our findings are relevant to perinatal outcomes given evidence linking sex steroid dysregulation and pregnancy complications [90], adverse birth outcomes [28], and adverse long-term child health outcomes [91]. Additional work is needed to replicate these findings in large, diverse populations with higher exposure to neighborhood disorder and violence. Additionally, there is a need for more research on the impact of neighborhood stress on individual-level stress measures in pregnancy as well as on the interactions between HPA and HPG axis activity in the maternal-placental-fetal unit.

### Electronic supplementary material

Below is the link to the electronic supplementary material.

Supplementary Material 1

## Data Availability

The datasets generated and/or analyzed during the current study are not publicly available because this is an ongoing active birth cohort, but are available from the corresponding author on reasonable request.

## Declarations

### Minor participation

Although the UPSIDE study followed the infants born from the cohort, the current analysis does not include data from these children.

## List of abbreviations

ACTH: Adrenocorticotropin hormone
AGD: Anogenital distance
BMI: Body Mass Index
CRH: Corticotropin releasing hormone
CSI: City Stress Inventory
E1: Estrone
E2: Estradiol
E3: Estriol
FT: Free Testosterone
HPA: Hypothalamic-pituitary-adrenal axis
HPG: Hypothalamic–pituitary–gonadal axis
LMM: Linear mixed models
LOD: Limit of detection
TT: Total Testosterone
