# Supplementary material for: Associations between neighborhood stress and maternal sex steroid hormones in pregnancy
Source: BMC Pregnancy Childbirth. 2023 Oct 16;23:730. doi: 10.1186/s12884-023-06043-0 (PMC10577914; doi:10.1186/s12884-023-06043-0)
Supplement: Supplementary file 1 — Supplementary Material 1 [file 12884_2023_6043_MOESM1_ESM.docx]

**Title: Associations Between Neighborhood Stress and Maternal Sex Steroid Hormones in Pregnancy**

*Authors*

Megan C. Hansel^a^*, Hannah R. Murphy^b^, Jessica Brunner^b^, Christina Wang^c^, Richard K. Miller^b^, Thomas G. O’Connor^d^, Emily S. Barrett^a^* and Zorimar Rivera-Núñez^a^*

*Affiliations*

^a^Department of Biostatistics and Epidemiology, Rutgers School of Public Health, Piscataway, NJ, USA

^b^Department of Obstetrics and Gynecology, University of Rochester, Rochester, NY, USA

^c^Clinical and Translational Science Institute, The Lundquist Institute at Harbor –UCLA Medical Center, Torrance, CA, USA

^d^Departments of Psychiatry, Psychology, Neuroscience, University of Rochester, NY, USA

*Denotes first author (MH) and shared senior author (EB, ZRN)

*Corresponding Author*

Zorimar Rivera-Núñez, PhD, MS

170 Frelinghuysen Rd

Piscataway, NJ 08854

e-mail: zr69@eohsi.rutgers.edu

**Supplemental Figure 1: Directed acyclic graph of the proposed relationship between variables**

**
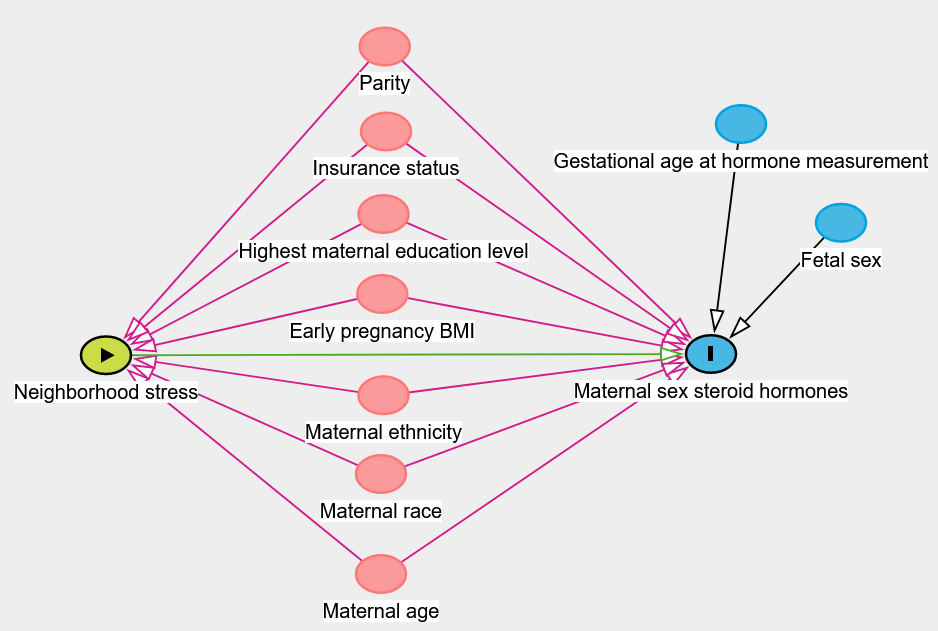
**

**Supplemental Figure 2: Pearson correlations between maternal sex steroid hormones in each trimester**

**
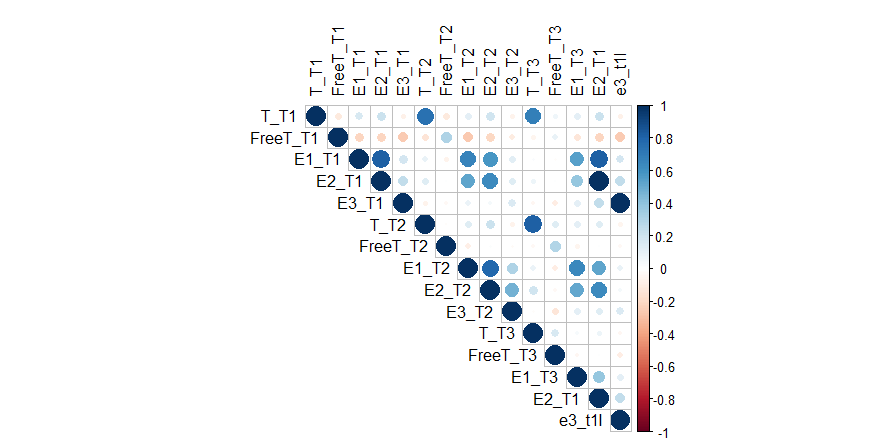
**

Abbreviations: T_T1- Total Testosterone Trimester 1, FreeT_T1- Free Testosterone Trimester 1, E1_T1- Estrone Trimester 1, E2_T1- Estradiol Trimester 1, E3_T1- Estriol Trimester 1, T_T2- Total Testosterone Trimester 2, FreeT_T2- Free Testosterone Trimester 2, E1_T2- Estrone Trimester 2, E2_T2- Estradiol Trimester 2, E3_T2- Estriol Trimester 2, T_T3- Total Testosterone Trimester 3, FreeT_T3- Free Testosterone Trimester 3, E1_T3- Estrone Trimester 3, E2_T3- Estradiol Trimester 3, E3_T3- Estriol Trimester 3

**Supplemental Table 1: Longitudinal models examining associations between neighborhood stress and maternal sex steroid hormone concentrations in pregnancy.**

|  | **Total Testosterone (TT, ng/dl)** | | **Free Testosterone (FT, ng/dl)** | | **Estrone (E1, pg/mL)** | | **Estradiol (E2, pg/mL)** | | **Estriol (E3, pg/mL)** | |
| --- | --- | --- | --- | --- | --- | --- | --- | --- | --- | --- |
|  | Crude  %Δ (95% CI) | Adjusted^1^  %Δ (95% CI) | Crude  %Δ (95% CI) | Adjusted^1^  %Δ (95% CI) | Crude  %Δ (95% CI) | Adjusted^1^  %Δ (95% CI) | Crude  %Δ (95% CI) | Adjusted^1^  %Δ (95% CI) | Crude  %Δ (95% CI) | Adjusted^1^  %Δ (95% CI) |
| **Neighborhood Disorder^2^ (n=258); REF=Q1** | | | | | | | | | | |
| Q2 | **45.02**  **(18.54, 77.43)** | **37.26**  **(13.16, 66.50)** | -1.21  (-7.65, 5.68) | -1.23  (-7.81, 5.82) | -0.51  (-20.16, 23.99) | -1.70  (-20.94, 22.24) | 4.81  (-8.06, 19.48) | 2.92  (-9.03, 16.44) | 7.26  (-4.13, 20.00) | 5.07  (-5.66, 17.02) |
| Q3 | **28.33**  **(5.52, 56.05)** | **22.15**  **(1.20, 47.46)** | -5.25  (-11.25, 1.14) | -5.56  (-11.68, 0.99) | 3.32  (-16.54, 27.90) | 1.02  (-18.31, 24.92) | 5.75  (-6.86, 20.07) | 4.31  (-7.50, 17.62) | 1.43  (-9.01, 13.07) | 1.24  (-8.82, 12.42) |
| Q4 | **49.80**  **(21.51, 84.65)** | **25.65**  **(1.63, 55.33)** | -3.36  (-9.89, 3.63) | -5.70  (-12.57, 1.70) | 0.83  (-19.74, 26.69) | -0.23  (-21.46, 26.74) | 5.04  (-8.30, 20.31) | 0.77  (-11.99, 15.37) | -1.83  (-12.59, 10.26) | 2.46  (-8.95, 15.29) |
| **Exposure to Violence^3^ (n=260); REF=none** | | | | | | | | | | |
| Any | **29.98**  **(9.15, 54.79)** | 5.26  (-12.61, 26.79) | -0.05  (-5.65, 5.88) | -2.42  (-8.58, 4.16) | 13.03  (-6.22, 36.22) | 12.06  (-8.64, 37.45) | **12.03**  **(0.18, 25.28)** | 5.11  (-6.50, 18.18) | -5.61  (-14.30, 3.97) | -3.94  (-13.24, 6.36) |

Abbreviations: %Δ- Percent Change, CI- Confidence Interval

^1^Adjusted for trimester, maternal age, early pregnancy BMI, gestational age deviation, fetal sex, parity, maternal race, maternal ethnicity, highest maternal education level, and Medicaid in pregnancy

^2^Q1 score=11, Q2 score= 12-13, Q3 score=14-17, Q4 score=18-42

^3^None score=7, any score >7

Bolded entries indicate p< 0.05

|  | **Total Testosterone (TT, ng/dl)** | | **Free Testosterone (FT, ng/dl)** | | **Estrone (E1, pg/mL)** | | **Estradiol (E2, pg/mL)** | | **Estriol (E3, pg/mL)** | |
| --- | --- | --- | --- | --- | --- | --- | --- | --- | --- | --- |
|  | Male | Female | Male | Female | Male | Female | Male | Female | Male | Female |
|  | %Δ (95% CI) | %Δ (95% CI) | %Δ (95% CI) | %Δ (95% CI) | %Δ (95% CI) | %Δ (95% CI) | %Δ (95% CI) | %Δ (95% CI) | %Δ (95% CI) | %Δ (95% CI) |
| **Neighborhood Disorder^2^; REF=Q1** | | | | | | | | | | |
| Q2 | **84.84**  **(37.78, 147.96)** | 15.17  (-11.50, 49.87) | -3.81  (-12.92, 6.26) | 0.70  (-9.02, 5.67) | -4.06  (-30.86, 33.12) | 4.86  (-22.14, 41.20) | 1.47  (-16.82, 23.79) | 8.45  (-7.37, 26.99) | 2.26  (-12.72, 19.83) | 4.19  (-10.57, 21.37) |
| Q3 | **56.82**  **(19.39, 105.96)** | 6.94  (-19.04, 41.26) | -5.83  (-14.13, 3.26) | -9.04  (-18.29, 1.25) | 9.50  (-19.21, 48.41) | -16.27  (-38.85, 14.65) | 11.19  (-7.55, 33.72) | -4.28  (-18.96, 13.05) | 1.57  (-12.30, 17.64) | 1.15  (-13.89, 18.83) |
| Q4 | **48.17**  **(8.13, 103.05)** | 12.34  (-16.51, 51.16) | -3.83  (-13.54, 6.97) | -8.35  (-18.25, 2.75) | 7.07  (-24.66, 52.15) | -11.96  (-37.04, 23.11) | 5.74  (-14.56, 30.88) | -5.46  (-20.83, 12.91) | -7.16  (-21.67, 10.03) | 9.47  (-7.81, 29.98) |
| **Exposure to Violence^3^; REF=none** | | | | | | | | | | |
| Any | 19.54  (-12.07, 62.50) | -2.19  (-22.81, 23.92) | 0.44  (-8.88, -10.72) | -3.63  (-12.11, 5.67) | 11.19  (-19.19, 53.00) | 4.34  (-20.11, 36.26) | 8.19  (-11.14, 31.73) | -0.11  (-13.39, 15.22) | -14.37  (-26.70, 0.03) | 1.46  (-11.48, 16.30) |

**Supplemental Table 2: Adjusted^1^ LMMs examining associations between neighborhood stress and maternal sex steroid concentrations by fetal sex.**

Abbreviations: %Δ- Percent Change, CI- Confidence Interval

^1^Adjusted for trimester, maternal age, early pregnancy BMI, gestational age deviation, parity, maternal race, maternal ethnicity, highest maternal education level, and Medicaid in pregnancy

^2^Male fetuses n=130 and female fetuses n=128. Q1 score=11, Q2 score= 12-13, Q3 score=14-17, Q4 score=18-42

^3^Male fetuses n=131 and female fetuses n=129. None score=7, any score >7

Bolded entries indicate p< 0.05

**Supplemental Table 3: Adjusted^1^ linear regression models examining neighborhood stress and hormone concentrations in 1^st^ and 2^nd^ trimesters.**

|  | **Total Testosterone (TT, ng/dl)** | | **Free Testosterone (FT, ng/dl)** | | **Estrone (E1, pg/mL)** | | **Estradiol (E2, pg/mL)** | | **Estriol (E3, pg/mL)** | |
| --- | --- | --- | --- | --- | --- | --- | --- | --- | --- | --- |
|  | T1 | T2 | T1 | T2 | T1 | T2 | T1 | T2 | T1 | T2 |
|  | %Δ  (95% CI) | %Δ  (95% CI) | %Δ  (95% CI) | %Δ  (95% CI) | %Δ  (95% CI) | %Δ  (95% CI) | %Δ  (95% CI) | %Δ  (95% CI) | %Δ  (95% CI) | %Δ  (95% CI) |
| **Neighborhood Disorder^2^; REF=Q1** | | | | | | | | | | |
| Q2 | **35.11**  **(9.61, 66.56)** | **40.40**  **(13.36, 73.89)** | -1.11  (-10.18, 8.87) | -2.74  (-11.34, 6.70) | -3.85  (-24.45, 22.37) | 0.91  (-19.96, 27.22) | -7.69  (-21.58, 8.67) | 2.62  (-10.18, 17.25) | -4.04  (-32.08, 35.59) | 7.94  (-5.20, 22.91) |
| Q3 | 20.06  (-2.11, 47.26) | 18.41  (-3.88, 45.89) | -4.41  (-12.97, 4.99) | -3.07  (-11.44, 6.09) | -5.33  (-25.17, 19.78) | -0.32  (-20.48, 24.95) | -9.07  (-22.45, 6.62) | 0.44  (-11.80, 14.37) | -0.20  (-28.78, 39.84) | 0.38  (-11.56, 13.93) |
| Q4 | 19.46  (-5.13, 50.43) | **27.71**  **(0.87, 61.68)** | -7.92  (-17.17, 2.37) | -3.80  (-13.13, 6.54) | -10.00  (-30.99, 17.38) | -4.93  (-26.36, 22.74) | -11.79  (-26.29, 5.58) | -4.37  (-17.43, 10.77) | 9.44  (-25.22, 60.17) | 3.45  (-10.35, 19.38) |
| **Exposure to Violence^3^; REF=none** | | | | | | | | | | |
| Any | 2.90  (-15.96, 25.99) | 3.57  (-15.71, 27.26) | -2.95  (-11.50, 6.44) | -0.08  (-8.48, 9.11) | 17.10  (-6.92, 47.31) | 4.05  (-16.33, 29.40) | 13.01  (-3.40, 32.20) | 4.31  (-8.01, 18.28) | -7.16  (-33.19, 29.02) | -5.12  (-16.12, 7.32) |

Abbreviations: T1- Trimester 1, T2- Trimester 2, %Δ-Percent Change, CI- Confidence Interval

^1^Models adjusted for maternal age, early pregnancy BMI, gestational age at blood sample, fetal sex, parity, maternal race, maternal ethnicity, highest maternal educational level, and Medicaid in pregnancy

^2^T1: n=256, T2: n=258. Q1 score=11, Q2 score= 12-13, Q3 score=14-17, Q4 score=18-42

^3^T1: n= 258, T2: n=260. None score=7, any score >7

Bolded entries indicate p< 0.05

**Supplemental Table 4: LMMs examining associations between neighborhood stress and hormone concentrations across pregnancy excluding participants who relocated.**

|  | **Total Testosterone (TT, ng/dl)** | | **Free Testosterone (FT, ng/dl)** | | **Estrone (E1, pg/mL)** | | **Estradiol (E2, pg/mL)** | | **Estriol (E3, pg/mL)** | |
| --- | --- | --- | --- | --- | --- | --- | --- | --- | --- | --- |
|  | Crude  %Δ (95% CI) | Adjusted^1^  %Δ (95% CI) | Crude  %Δ (95% CI) | Adjusted^1^  %Δ (95% CI) | Crude  %Δ (95% CI) | Adjusted^1^  %Δ (95% CI) | Crude  %Δ (95% CI) | Adjusted^1^  %Δ (95% CI) | Crude  %Δ (95% CI) | Adjusted^1^  %Δ (95% CI) |
| **Neighborhood Disorder^2^ (n=234); REF=Q1** | | | | | | | | | | |
| Q2 | **54.31**  **(25.11, 90.33)** | **41.92**  **(15.92, 73.74)** | -0.85  (-7.48, 6.25) | -1.25  (-8.07, 6.08) | 1.23  (-19.52, 27.32) | -1.70  (-21.79, 23.55) | 8.16  (-5.58, 23.90) | 4.79  (-7.90, 19.23) | 11.03  (-1.24, 24.81) | 7.68  (-3.69, 20.38) |
| Q3 | **34.08**  **(8.98, 64.97)** | **26.55**  **(3.56, 54.65)** | -6.60  (-12.77, 0.00) | **-7.20**  **(-13.55, -0.38)** | 6.81  (-14.84, 33.95) | 5.10  (-16.21, 31.81) | 7.27  (-6.20, 22.67) | 6.82  (-6.00, 21.39) | 5.76  (-5.77, 18.71) | 4.81  (-6.14, 17.03) |
| Q4 | **57.66**  **(26.16, 97.03)** | 25.21  (-0.50, 57.57) | -3.82  (-10.64, 3.51) | -6.36  (-13.67, 1.58) | 5.86  (-17.02, 35.05) | 4.60  (-19.32, 35.61) | 9.34  (-5.35, 26.30) | 3.48  (-10.63, 19.81) | 0.11  (-11.58, 13.35) | 2.80  (-9.42, 16.67) |
| **Exposure to Violence^3^ (n=236); REF=none** | | | | | | | | | | |
| **Any** | **34. 59**  **(11.74, 62.14)** | 6.40  (-13.05, 30.20) | 0.22  (-5.67, 6.47) | -1.71  (-8.38, 5.45) | 13.62  (-6.80, 38.51) | 15.90  (-7.04, 44.51) | **16.09**  **(3.19, 30.60)** | 11.15  (-2.01, 26.07) | -3.46  (-12.93, 7.05) | -1.13  (-11.35, 10.28) |

Abbreviations: %Δ- Percent Change, CI- Confidence Interval

^1^Adjusted for trimester, maternal age, early pregnancy BMI, gestational age deviation, fetal sex, parity, maternal race, maternal ethnicity, highest maternal education level, and Medicaid in pregnancy

^2^Q1 score=11, Q2 score= 12-13, Q3 score=14-17, Q4 score=18-42

^3^None score=7, any score >7

Bolded entries indicate p< 0.05

**Supplemental Table 5: Adjusted^1^ linear regression models examining neighborhood stress and hormone concentrations excluding participants who relocated.**

|  | **Total Testosterone (TT, ng/dl)** | | | **Free Testosterone (FT, ng/dl)** | | | **Estrone (E1, pg/mL)** | | | **Estradiol (E2, pg/mL)** | | | **Estriol (E3, pg/mL)** | | |
| --- | --- | --- | --- | --- | --- | --- | --- | --- | --- | --- | --- | --- | --- | --- | --- |
|  | T1 | T2 | T3 | T1 | T2 | T3 | T1 | T2 | T3 | T1 | T2 | T3 | T1 | T2 | T3 |
|  | %Δ  (95% CI) | %Δ  (95% CI) | %Δ  (95% CI) | %Δ  (95% CI) | %Δ  (95% CI) | %Δ  (95% CI) | %Δ  (95% CI) | %Δ  (95% CI) | %Δ  (95% CI) | %Δ  (95% CI) | %Δ  (95% CI) | %Δ  (95% CI) | %Δ  (95% CI) | %Δ  (95% CI) | %Δ  (95% CI) |
| **Neighborhood Disorder^2^; REF=Q1** | | | | | | | | | | | | | | | |
| Q2 | **37.40**  **(10.29, 71.19)** | **46.86 (17.12, 84.16)** | **45.16 (15.62, 82.24)** | -1.10  (-12.06, 7.35) | -2.47  (-11.33, 7.28) | 0.19  (-12.17, 8.03) | -6.07  (-27.14, 21.09) | 3.16  (-19.09, 31.53) | -5.88  (-26.18, 19.99) | -7.85  (-22.48, 9.55) | 5.35  (-8.32, 21.06) | 3.50  (-9.28, 26.55) | -1.88  (-31.74, 41.03) | 10.42  (-3.53, 26.39) | 5.52  (-8.14, 21.21) |
| Q3 | **24.70**  **(0.29, 55.06)** | 21.59  (-2.82, 52.13) | **33.81**  **(6.92, 67.47)** | -7.32  (-16.12, 2.39) | -4.60  (-13.19, 4.84) | -8.66  (-17.72, 1.41) | -3.91  (-25.29, 23.60) | 6.78  (-16.05, 35.82) | 11.54  (-12.22, 41.72) | -8.79  (-23.16, 8.26) | 4.22  (-9.18, 19.59) | 10.08  (-3.34, 25.37) | 11.20  (-22.39, 59.31) | 3.14  (-9.77, 17.89) | 5.18  (-8.26, 20.58) |
| Q4 | 17.52  (-8.49, 50.93) | 24.50  (-3.75, 61.04) | **36.51**  **(5.53, 76.59)** | -8.79  (-18.65, 2.28) | -4.72  (-14.50, 6.18) | -4.51  (-15.30, 7.66) | -7.90  (-31.02, 22.97) | 5.12  (-20.25, 38.56) | 16.81  (-11.26, 53.75) | -10.19  (-26.23, 9.34) | 0.73  (-14.00, 17.97) | 7.62  (-7.29, 24.93) | 7.46  (-28.88, 62.39) | 5.37  (-9.62, 22.86) | -0.57  (-15.00, 16.31) |
| **Exposure to Violence^3^; REF=none** | | | | | | | | | | | | | | | |
| Any | 4.56  (-16.02, 30.19) | 3.94  (-17.00, 30.15) | 12.07  (-10.56, 40.42) | -2.84  (-12.06, 7.35) | 1.36  (-7.67, 11.26) | -2.59  (-12.17, 8.03) | 23.72  (-3.44, 58.51) | 9.54  (-13.38, 38.51) | 14.81  (-9.36, 45.42) | **20.84**  **(1.96, 43.22)** | 11.44  (-2.55, 27.45) | 11.08  (-2.51, 26.55) | -13.49  (-39.32, 23.35) | -3.40  (-15.35, 10.23) | 2.41  (-10.64, 17.37) |

Abbreviations: T1- Trimester 1, T2- Trimester 2, T3- Trimester 3, %Δ-Percent Change, CI- Confidence Interval

^1^Models adjusted for maternal age, early pregnancy BMI, gestational age at blood sample, fetal sex, parity, maternal race, maternal ethnicity, highest maternal educational level, and Medicaid in pregnancy

^2^T1: n=232, T2: n=234, T3: n=233. Q1 score=11, Q2 score= 12-13, Q3 score=14-17, Q4 score=18-42

^3^T1: n= 234, T2: n=236, T3: n=235. None score=7, any score >7

Bolded entries indicate p< 0.05
